# Blue Light Enhances Cadmium Tolerance of the Aquatic Macrophyte *Potamogeton crispus*

**DOI:** 10.3390/plants12142667

**Published:** 2023-07-17

**Authors:** Shanwei Wang, Liyuan Wang, Miao Zhang, Wei Li, Zuoming Xie, Wenmin Huang

**Affiliations:** 1 School of Environmental Studies, China University of Geosciences, Wuhan 430074, China; 2 Aquatic Plant Research Center, Wuhan Botanical Garden, Chinese Academy of Sciences, Wuhan 430074, China; 3 School of Ecology and Environment, Tibet University, Lhasa 850000, China; 4 Hubei Key Laboratory of Wetland Evolution & Ecological Restoration, Wuhan Botanical Garden, Chinese Academy of Sciences, Wuhan 430074, China

**Keywords:** light quality, *Potamogeton crispus*, cadmium tolerance, antioxidant enzymes, blue light, red light

## Abstract

Cadmium (Cd) is highly toxic and widely distributed in aquatic systems due to its high solubility and mobility in water, which can severely inhibit the survival of aquatic macrophytes. The phytotoxicity of Cd depends on environmental factors; however, it remains unclear whether and how light quality affects its toxicity on aquatic macrophytes. In this study, we investigated the effects of Cd on aquatic macrophytes *Potamogeton crispus* under different light qualities (white, blue, and red light). We evaluated morphological and photo-physiological traits, as well as the cellular antioxidant defense system. Our findings indicate that *P. crispus* under Cd stress showed notable damage in leaf morphology, decreased photosynthetic efficiency, inhibited HCO_3_^−^ uptake, and reduced antioxidant enzyme activities, as well as oxidative damage indicated by MDA accumulation and superoxide (O_2_^−^) overproduction. However, compared with white or red light under Cd stress, blue light reduced structural damage and oxidative stress caused by Cd while increasing pigment synthesis and photosynthetic efficiency, as well as increasing ascorbate peroxidase (APX) activity. In conclusion, the changes induced by blue light in *P. crispus*’s photosynthesis and antioxidant system strengthen its tolerance to Cd. Further research on signal transmission in relation to light quality in Cd-exposed aquatic plants is still needed.

## 1. Introduction

Ecological problems are increasingly serious worldwide, with the degradation of aquatic plants being a particular concern for researchers [1]. Industrialization has led to rising levels of pollutants, including heavy metals, in the water near human settlements [2]. This pollution may be contributing to the decline of aquatic macrophytes [3]. Cadmium (Cd), a heavy metal widely distributed in aquatic systems, enters water bodies through the use of phosphorus fertilizers and industrial discharge [4]. The high mobility of Cd in plant systems is especially worrying, as it can lead to food chain contamination and potentially harm human health [5].

Research on Cd-induced phytotoxicity has been extensive, covering both terrestrial and aquatic plants. Cd exposure can reduce chlorophyll content, inhibit photosynthesis, damage cell structure, disrupt ion metabolism, and affect normal physiological status, eventually leading to plant death [6]. However, plants have evolved detoxification mechanisms to counteract the oxidative damage caused by Cd toxicity [7]. Studies have observed significant phytochelatin synthesis in Cd-exposed *Pistia stratiotes*, while other plants, such as *Eichhornia crassipes* and *Cabomba caroliniana,* rely on antioxidant defenses to tolerate Cd-induced toxicity [8,9]. Vital antioxidant enzymes such as superoxide dismutase (SOD), catalase (CAT), peroxidase (POD), and glutathione reductase (GR) are essential components of the antioxidant system in plants [10]. Additionally, ascorbate peroxidase (APX) is a hydrogen peroxide scavenging enzyme specific to plants and algae that plays a critical role in protecting chloroplasts and other cellular components from damage caused by H_2_O_2_ and hydroxyl radicals (·OH) [11].

In addition to the negative impacts of heavy metals, environmental changes, such as eutrophication and water level fluctuations, can lead to reduced underwater light conditions, including changes in light intensity and quality, which may also be a core factor contributing to the decline of aquatic macrophytes [12,13]. Light quality is an essential factor of light conditions that can be perceived by plants through various photoreceptors, including cryptochrome, phytochrome, and phototropin [14]. It can affect the growth, development, physiological metabolism, and stress responses of plants [14,15]. In a water column, the range of light wavelengths changes from a nearly complete spectrum at the interface between air and water to a restricted, predominantly blue spectrum in deeper layers. This phenomenon is primarily caused by the absorption of dissolved substances and suspended particles in the water [16]. Red and blue light are the primary energy sources for photosynthetic CO_2_ assimilation in plants [14]. Numerous studies have demonstrated that terrestrial plants exhibit species-specific responses to different light qualities [17,18]. Pettai et al. (2005) [19] discovered that red light can enhance the photosynthetic activity of sunflowers and beans, primarily by supporting oxygen evolution [19]. Similarly, Muneer et al. (2014) [20] reported that increasing blue light intensity can boost the biomass and photosynthetic metabolism of *Lactuca sativa* L. [20]. However, research on the response of aquatic plants to light quality remains limited [21]. Our recent study investigated the effects of monochromatic blue and red light on photomorphogenesis and photosynthetic physiology in the aquatic macrophyte *Ottelia alismoides*, revealing differential responses to these light qualities [13].

Our previous study on the aquatic plant *Potamogeton crispus* found that the toxic effects of Cd were strongly dependent on light intensity [22]. However, the effect of light quality on Cd toxicity in aquatic plants remains unknown. Recent research has revealed that blue and red light can have opposing effects on the response of terrestrial plant cucumber to Cd-induced stress by regulating photosynthetic metabolism and antioxidant system response [23]. Nonetheless, there is limited research on how light quality affects the response of aquatic macrophytes to Cd-induced toxicity. *P. crispus* is a submerged macrophyte that is widely distributed and grows rapidly. It has been reported to accumulate Cd more easily than other aquatic plants [24]. It is commonly found in shallow waters, such as freshwater lakes, ponds, rivers, and streams, but it can also invade deep-water areas and grow up to 4 m deep [25]. Therefore, *P. crispus* in different water layers can receive varying light qualities in its in situ habitat. The aim of our study was to investigate how different light qualities affect *P. crispus* under Cd stress and elucidate the underlying mechanisms. Red and blue light are visible light wavelengths that primarily influence plant development and growth. For this purpose, *P. crispus* plants were exposed to Cd under white, red, and blue light provided by light-emitting diodes (LEDs), which offer continuous stability at specific wavelengths [13]. We evaluated the impact of Cd toxicity on *P. crispus* plants exposed to different light qualities by observing leaf morphology and organelle ultrastructure, as well as measuring photosynthesis and antioxidant system-related parameters. The findings of this study not only expand our understanding of how light quality can induce tolerance to Cd stress in aquatic plants but also provide insight into how LEDs can regulate the responses of aquatic plants to Cd stress. This knowledge can facilitate the restoration of aquatic vegetation in metal-polluted water bodies using LEDs.

## 2. Results

### 2.1. Leaf Morphology and Anatomic Structure

**Figure 1:**
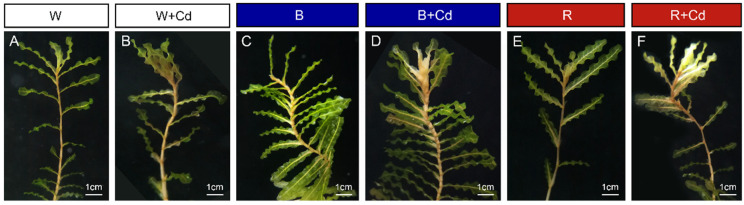
The *P. crispus* phenotype treated with or without 50 μM Cd under different light qualities. (**A**) No Cd under white light; (**B**) 50 µM Cd treatment under white light; (**C**) no Cd under blue light; (**D**) 50 µM Cd treatment under blue light; (**E**) no Cd under red light; (**F**) 50 µM Cd treatment under red light. Scale bar = 1 cm.

**Figure 2:**
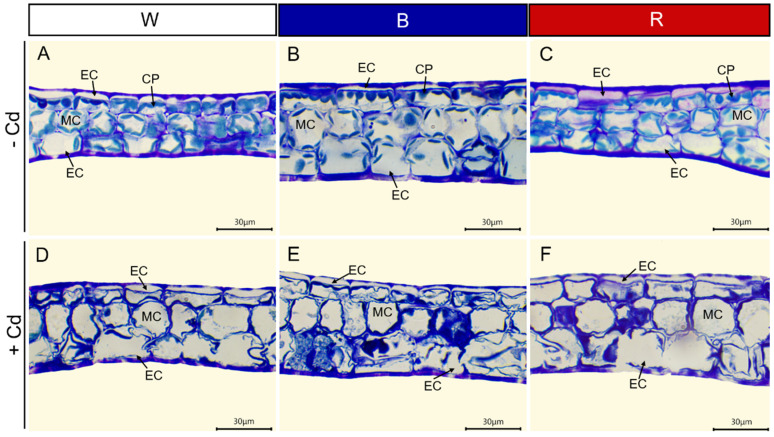
The effect of light quality and Cd on the leaf anatomy of *P. crispus*. (**A**) No Cd under white light; (**B**) no Cd under blue light; (**C**) no Cd under red light; (**D**) 50 µM Cd treatment under white light; (**E**) 50 µM Cd treatment under blue light; (**F**) 50 µM Cd treatment under red light. EC, epidermal cell; CP, chloroplast; MC, mesophyll cell. Scale bar = 30 µm.

Regardless of light quality, Cd toxicity induced chlorosis and necrosis of *P. crispus* leaves. However, the application of blue light reduced the toxic symptoms (Figure 1). To understand the effect of different light qualities on the leaf anatomy of *P. crispus* under Cd stress, transverse sections were observed. Under Cd-free conditions, *P. crispus* leaves comprised an upper and lower epidermis, as well as a layer of stacked mesophyll cells (Figure 2). Abundant chloroplasts were present in both the epidermal and mesophyll cells in all three light qualities (Figure 2A–C). After Cd exposure, deformed chloroplasts and cracks in the cell membrane were observed in W + Cd-treated *P. crispus* leaves (Figure 2D). A similar type of damage was also observed in B + Cd-treated *P. crispus* leaves (Figure 2E). However, more serious damage to the anatomic structure was present in R + Cd groups (Figure 2F).

### 2.2. Ultrastructure of Chloroplasts and Mitochondria

**Figure 3:**
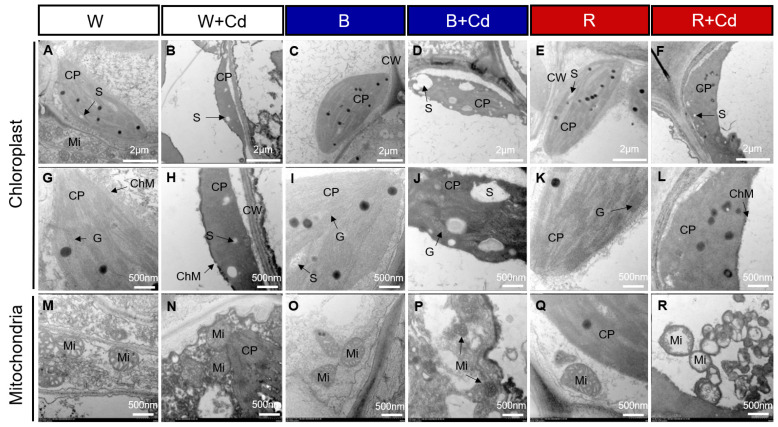
Transmission electron microscopy (TEM) of the chloroplast and mitochondria ultrastructure in the leaves of *P. crispus* treated without or with Cd under different light qualities. (**A**,**G**,**M**) No Cd under white light; (**B**,**H**,**N**) 50 µM Cd treatment under white light; (**C**,**I**,**O**) no Cd under blue light; (**D**,**J**,**P**) 50 µM Cd treatment under blue light; (**E**,**K**,**Q**) no Cd under red light; (**F**,**L**,**R**) 50 µM Cd treatment under red light. CP, chloroplast; Mi, mitochondria; ChM, chloroplast membrane; G, grana; S, starch; CW, cell wall. Scale bar in A–F = 2 µm; scale bar in G–R = 500 nm. W, W + Cd, B, B + Cd, R, and R + Cd indicate the treatment of no Cd under white light, 50 µM Cd exposure under white light, no Cd under blue light, 50 µM Cd exposure under blue light, no Cd under red light, and 50 µM Cd exposure under red light, respectively.

Figure 3 shows the chloroplast ultrastructure in *P. crispus* plants treated with and without Cd under different light qualities. Under Cd-free conditions (Figure 3A,C,E,G,I,K), the chloroplasts of *P. crispus* were well-developed and presented a regular oval shape. When *P. crispus* plants were exposed to Cd under W or R, the grana and thylakoids were indistinct, thylakoids tended to disintegrate, and their structure was blurred (Figure 3B,F,H,L). However, the relatively better physical condition of thylakoids was observed in the B + Cd-exposed *P. crispus* (Figure 3D,J).

Regarding mitochondria, under Cd-free conditions, lots of mitochondria with high electronic densities and abundant cristae were distributed in the cells of *P. crispus* leaves, regardless of light quality (Figure 3M,O,Q). After Cd exposure, the mitochondria were damaged, with vague and much fewer cristae in W + Cd-treated *P. crispus* leaves compared to W groups (Figure 3N). In R + Cd groups, badly damaged mitochondria with less electron density and fewer cristae were observed (Figure 3R). However, a much better ultrastructure of mitochondria with relatively high electronic densities and clear cristae were observed in B + Cd-treated *P. crispus* leaves (Figure 3P).

### 2.3. Pigment Content, Chlorophyll Fluorescence, and Photosynthetic Rate

**Figure 4:**
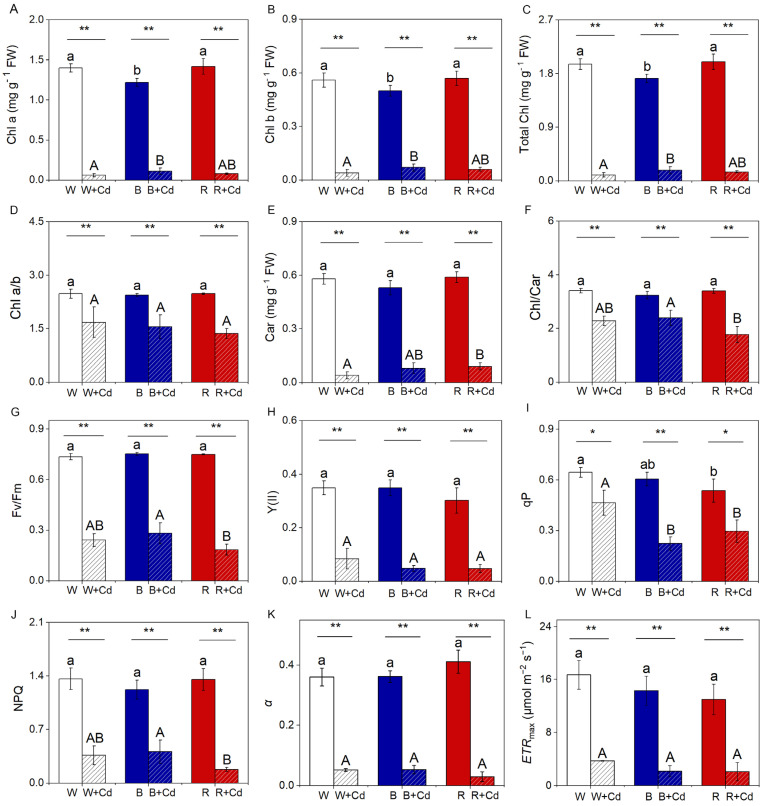
The effect of light quality and Cd on pigment content and chlorophyll fluorescence of *P. crispus*. (**A**) Chl a; (**B**) Chl b; (**C**) total Chl; (**D**) Chl a/b; (**E**) Car; (**F**) Chl/Car; (**G**) Fv/Fm; (**H**) Y(II); (**I**) qP; (**J**) NPQ; (**K**) α; (**L**) *ETR*_max_. Data are presented as mean ± SD (*n* = 4). The statistical differences were tested using independent sample *t*-tests and ANOVA followed by Duncan’s and Tukey’s post-hoc tests. The statistic above the horizontal line compares the leaves treated without Cd and with Cd grown under the same light quality (** *p* < 0.01, * *p* < 0.05). Data with different lowercases (a, b) are significantly different among different light qualities for no Cd-treated *P. crispus* leaves (*p* < 0.05). Data with different uppercase letters (A, B) are significantly different among different light qualities for Cd-exposed *P. crispus* leaves (*p* < 0.05). W, W + Cd, B, B + Cd, R, and R + Cd indicate the treatment of no Cd under white light, 50 µM Cd exposure under white light, no Cd under blue light, 50 µM Cd exposure under blue light, no Cd under red light, and 50 µM Cd exposure under red light, respectively.

Without Cd treatment, B significantly decreased the levels of Chl a, b, and total Chl in *P. crispus* compared to W and R (*p* < 0.05, Figure 4A–C). However, there was no significant difference between W and R for these parameters (*p* > 0.05, Figure 4A–C). Cd pollution led to a significant reduction in Chl a, b, and total Chl in W-grown *P. crispus* (*p* < 0.05, Figure 4A–C). However, after Cd exposure, B markedly increased the levels of these parameters compared to W + Cd groups (*p* < 0.05, Figure 4A–C). No obvious difference in Chl a, b, and total Chl was detected between W + Cd and R + Cd groups (*p* > 0.05, Figure 4A–C). Chl a/b in Cd-treated *P. crispus* plants was significantly lower than Cd-free conditions, regardless of light quality (*p* < 0.05, Figure 4D). As for Car content and the Chl/Car ratio, there was no significant difference among the *P. crispus* leaves exposed to different light qualities under Cd-free conditions (*p* > 0.05, Figure 4E,F). Compared to W + Cd groups, the Car content was significantly reduced in R + Cd groups (*p* < 0.05, Figure 4E). However, B-grown *P. crispus* plants exposed to Cd increased the Car content but not significantly when compared with W + Cd plants (*p* > 0.05, Figure 4E). Furthermore, the ratio of Chl/Car in B + Cd groups was significantly higher than R + Cd (*p* < 0.05, Figure 4F) but was comparable to W + Cd (*p* > 0.05, Figure 4F). There was a significant interaction between Cd and light quality on the content of Chl a (*p* < 0.001), Chl b (*p* < 0.01), total Chl (*p* < 0.01), and Chl/Car (*p* < 0.05, Table 1).

Under Cd-free conditions, no significant difference in Fv/Fm, NPQ, Y(II), qP, *ETR*_max_, and α was found among the *P. crispus* leaves exposed to different light qualities (*p* > 0.05, Figure 4G–L), except that W significantly increased qP when compared to R (*p* < 0.05, Figure 4I), while being comparable to B (*p* > 0.05, Figure 4I). Cd exposure significantly decreased the levels of Fv/Fm, Y(II), qP, NPQ, α, and *ETR*_max_ in W-grown *P. crispus* when compared to the Cd-free group (*p* < 0.05, Figure 4G–L). Moreover, R further significantly decreased Fv/Fm, qP, and NPQ in R + Cd groups compared to W + Cd-treated *P. crispus* plants (*p* < 0.05, Figure 4G,I,J). Conversely, the levels of the mentioned parameters were comparable in B + Cd groups relative to W + Cd treatment groups (*p* > 0.05, Figure 4G,H,J–L), except for qP, which was significantly lower in B + Cd groups compared to W + Cd groups (*p* < 0.05, Figure 4I). Statistical analysis showed that Cd had a greater effect on chlorophyll and chlorophyll fluorescence than light quality (Table 1). Furthermore, the interaction between Cd and light quality significantly affected qP and α (*p* < 0.05, Table 1). Regarding the photosynthetic rate, after Cd exposure, the O_2_ evolution rate was only detected in B-grown *P. crispus* plants (Table 2).

### 2.4. HCO_3_^−^ Uptake

At the end of pH-drift, there was no significant difference in the final pH among the *P. crispus* leaves exposed to different light quality under Cd-free conditions (*p* > 0.05, Table 3). Cd exposure significantly decreased the final pH when grown under R compared to B-grown groups (*p* < 0.05, Table 3). However, B-grown *P. crispus* plants exposed to Cd increased the final pH, but not significantly when compared with W + Cd plants (*p* > 0.05, Table 3). Moreover, the final pH in Cd-treated groups was significantly lower than Cd-free conditions, regardless of light quality (*p* < 0.05, Table 3). Under either Cd-free or Cd-exposed conditions, there was no significant difference in ALK and C_T_/ALK among the *P. crispus* leaves exposed to different light qualities (*p* > 0.05, Table 3). However, after Cd exposure, the C_T_/ALK was significantly higher than in the Cd-free groups (*p* < 0.05, Table 3) and was close to 1, regardless of the light quality. Furthermore, after Cd exposure, the concentration of C_T_, CO_2_, and HCO_3_^−^ in the solution was significantly higher than in Cd-free conditions, regardless of light quality (*p* < 0.05, Table 3). Moreover, the CO_2_ concentration in B + Cd was significantly lower than R + Cd (*p* < 0.05, Table 3) and was slightly lower compared to W + Cd (*p* > 0.05, Table 3). Statistical analysis showed that Cd affected the final pH, CO_2_, HCO_3_^−^, and C_T_/ALK more significantly than light quality (Table 1), while no significant interaction between the two factors was found to affect the above-mentioned parameters (*p* > 0.05, Table 1).

### 2.5. MDA Content, In Situ O_2_^.−^ Accumulation, and Antioxidant Enzyme Activity

**Figure 5:**
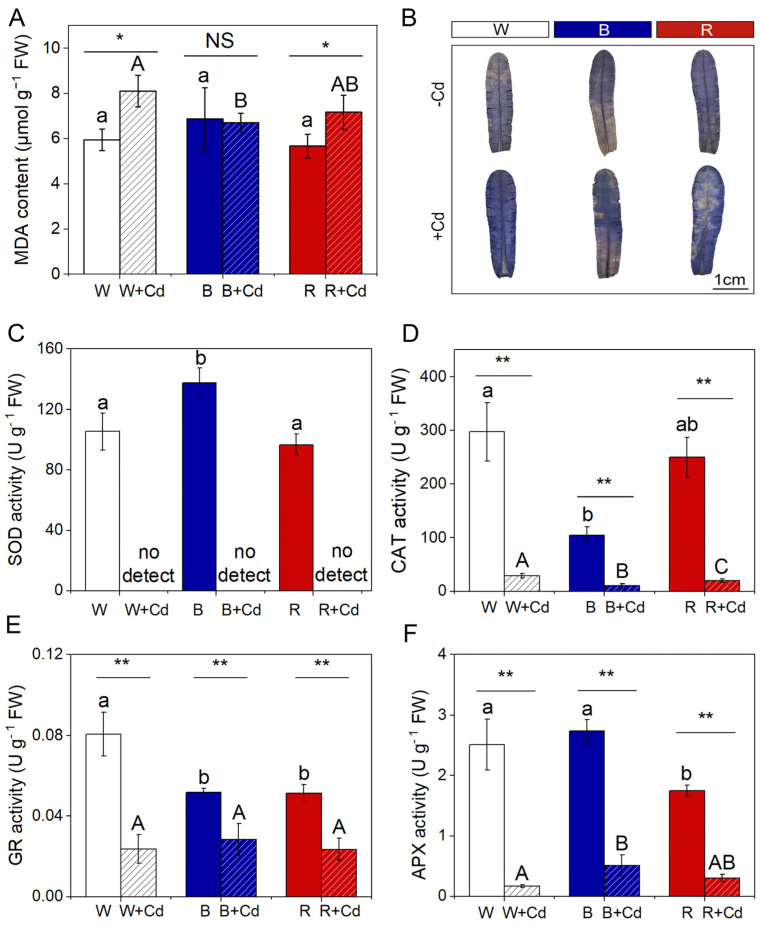
The effect of light quality and Cd on MDA content, in situ O_2_^.−^ accumulation, and antioxidant enzyme activity of *P. crispus*. (**A**) MDA content; (**B**) histochemical staining of O_2_^.−^ by NBT in *P. crispus* leaves; (**C**) SOD activity; (**D**) CAT activity; (**E**) GR activity; (**F**) APX activity. Data are presented as mean ± SD (*n* = 4) using independent sample *t*-tests and ANOVA followed by Duncan’s and Tukey’s post-hoc tests to test the statistical differences. The statistic above the horizontal line compares the leaves treated without Cd and with Cd grown under the same light quality (NS not significant, * *p* < 0.05, ** *p* < 0.01). Data with different lowercases (a, b) are significantly different among different light qualities for no Cd-treated *P. crispus* leaves (*p* < 0.05). Data with different uppercase letters (A, B) are significantly different among different light qualities for Cd-exposed *P. crispus* leaves (*p* < 0.05). W, W + Cd, B, B + Cd, R, and R + Cd indicate the treatment of no Cd under white light, 50 µM Cd exposure under white light, no Cd under blue light, 50 µM Cd exposure under blue light, no Cd under red light, and 50 µM Cd exposure under red light, respectively.

MDA content did not significantly differ among *P. crispus* leaves exposed to different light qualities under Cd-free conditions (*p* > 0.05, Figure 5A). Cd exposure significantly elevated the levels of MDA in W + Cd- and R + Cd-treated *P. crispus* compared to Cd-free groups (*p* < 0.05, Figure 5A). However, there was no significant difference in MDA content between B and B + Cd groups (*p* > 0.05, Figure 5A). Furthermore, the MDA content in B + Cd groups was significantly lower than W + Cd (*p* < 0.05, Figure 5A), while being comparable to R + Cd conditions (*p* > 0.05, Figure 5A). Cd affected the synthesis of MDA more significantly than light quality (Table 1). Moreover, there was a significant interaction between Cd and light quality on MDA content, which induced the most production of MDA under white light with Cd exposure (*p* < 0.05, Table 1).

Distinct overproduction of O_2_^.−^ induced by Cd in *P. crispus* leaves was verified by a histochemical method with NBT. Without Cd exposure, the *P. crispus* leaves were slightly stained by NBT under all the light qualities (Figure 5B). After Cd exposure, the *P. crispus* leaves under W were stained blue by NBT (Figure 5B). The leaves treated in B + Cd and R + Cd presented a weaker and stronger NBT stain compared to W + Cd-treated leaves, respectively (Figure 5B).

Under Cd-free conditions, light quality significantly affected the activity of SOD and CAT enzymes in *P. crispus*. Specifically, B increased SOD activity compared to W and R (*p* < 0.05, Figure 5C), while B decreased CAT activity compared to W (*p* < 0.05, Figure 5D). However, there was no significant difference in SOD and CAT activity between W and R (*p* > 0.05, Figure 5C,D). After Cd exposure, SOD activity was undetectable in *P. crispus* under all light quality conditions (Figure 5C), while CAT activity significantly varied with light quality (*p* < 0.05, Figure 5D). The plants treated with Cd and grown under W had the highest CAT activity, followed by the R + Cd- and B + Cd-treated plants (*p* < 0.05, Figure 5D). The interaction between Cd and light quality significantly affected SOD and CAT activity (*p* < 0.05, Table 1). For GR activity, both B and R significantly decreased their activity compared to W-grown *P. crispus* under Cd-free conditions (*p* < 0.05, Figure 5E). After Cd exposure, there was a significant decrease in GR activity in *P. crispus* leaves compared to the control group under all light quality conditions (*p* < 0.05, Figure 5E). However, there was no significant difference in GR activity among the treatments of W + Cd, B + Cd, and R + Cd (*p* > 0.05, Figure 5E). The interaction between Cd and light quality significantly influenced GR activity (*p* < 0.05, Table 1). Regarding APX activity, R significantly decreased its activity compared to W and B under Cd-free conditions (*p* < 0.05, Figure 5F). After Cd treatment, APX activity significantly decreased in *P. crispus* leaves compared to the control group, regardless of light quality (*p* < 0.05, Figure 5F). However, the APX activity in B + Cd groups significantly increased compared to W + Cd groups (*p* < 0.05, Figure 5F), while no significant difference was present between W + Cd and R + Cd groups (*p* > 0.05, Figure 5F). The interaction between Cd and light quality significantly influenced APX activity in *P. crispus* (*p* < 0.05, Table 1).

## 3. Discussion

Cd exposure can cause significant phytotoxicity in aquatic plants, leading to damaged anatomic structures, deformed organelle ultrastructure, decreased photosynthetic pigment biosynthesis, and reduced photosynthetic efficiency [9,22,26]. Furthermore, it has been confirmed that Cd can affect the functionality of CO_2_-concentrating mechanisms (CCMs) in aquatic plants [27]. However, little research has been conducted on the effects of light quality on aquatic plants under Cd stress, and the potential regulatory mechanisms are still unknown. In this study, we demonstrated that blue and red light can regulate tolerance to Cd in the aquatic plant *P. crispus* by inducing different reactions in the photosynthetic and antioxidant systems.

### 3.1. The Effect of Light Qualities on Leaf Anatomy and the Ultrastructure of Chloroplasts and Mitochondria of P. crispus under Cd Stress

In this study, Cd treatment significantly damaged the leaf anatomic structure and ultrastructure of chloroplasts and mitochondria in *P. crispus* leaves. The leaf anatomy exhibited clear evidence of toxicity effects induced by Cd exposure in *P. crispus*, including blurry boundaries between cells and broken cell membranes. These results are consistent with our previous studies on *P. crispus* and *Ottelia alismoides* [22,27]. Ultrastructure observation indicated that Cd treatment induced damage to chloroplasts and mitochondria, altering their shape and causing swelling of the thylakoid layer/mitochondrial cristae in *P. crispus*, regardless of light quality. Mitochondria, essential organelles found in most eukaryotic cells, play a crucial role in plant physiology, particularly in photosynthesis. Our results are in agreement with the findings of earlier research on different plant species [22,23,28]. Interestingly, B treatment relieved the damage to the ultrastructure of chloroplasts and mitochondria in *P. crispus* under Cd exposure. However, compared to W treatment during Cd stress, R further aggravated the damage induced by Cd stress. The findings of this study demonstrate that R increased the sensitivity of *P. crispus* plants to Cd, while B enhanced Cd tolerance in these plants. Similar conclusions were drawn by Guo et al. (2022) [23], who found that compared to red light, blue light reduced chlorosis and decreased chloroplast ultrastructure damage induced by Cd in cucumbers [23]. Reducing Cd accumulation is often linked to the alleviation of Cd toxicity in plants [29]. A study on Cd-treated cucumber seedlings found that blue light significantly decreased the expression of Cd uptake and transport genes, including *IRT1*, *NRAMP1*, and *HMA3*, leading to lower Cd accumulation [23].

### 3.2. The Effect of Light Qualities on Pigment, Photochemistry, and HCO_3_^−^ Uptake of P. crispus under Cd Stress

The normal functioning of a biological system depends on its structural integrity. The normal structure of chloroplasts is crucial for photosynthesis, as it affects the absorption of light and the capacity of electron transport due to the distribution of chlorophyll in the granum thylakoids [30,31]. Thus, the destruction of chloroplast structure induced by Cd ultimately leads to decreased biosynthesis of chlorophyll, reduced photosynthetic rate, and damaged photosynthetic efficiency [22]. In this study, we found that Cd exposure significantly decreased the content of chlorophyll and carotenoids, regardless of light quality. However, B was more effective in maintaining a relatively high level of chlorophyll in *P. crispus* plants compared to W treatment during Cd exposure. Thus, the application of blue light as a light condition may alleviate the harmful effects caused by Cd and maintain the survival of *P. crispus* by increasing the availability of pigments for tolerance to Cd-induced toxicity. The increased synthesis of pigments is believed to be a protective mechanism against metal toxicity, as pigments can act as antioxidants and help to scavenge harmful reactive oxygen species in plant tissues [32].

Chlorophyll fluorescence analysis has been widely used to investigate the defense mechanisms of the photosynthetic apparatus under various stresses. In this study, we evaluated the effects of different light qualities on the photosynthetic performance in vivo in *P. crispus* under Cd stress based on chlorophyll fluorescence measurements. Generally, Cd-stressed plants have a significantly lower Fv/Fm value than non-stressed plants [22], which is closely related to the photoinhibition of PSII [33]. In the present study, Cd significantly decreased Fv/Fm and the yield of PSII in *P. crispus*, regardless of light quality, indicating that electron transport and photosynthetic efficiency were both damaged by Cd toxicity, which was consistent with previous results [22,34]. However, the significantly higher level of Fv/Fm in *P. crispus* grown with B + Cd compared to R + Cd implies that *P. crispus* exposed to Cd suffered from a less stressful environment when grown under blue light. Furthermore, the significantly increased NPQ under B + Cd suggests an increase in the thermal dissipation of excess light energy. Increased NPQ has also been reported in the Cd-exposed aquatic plant *Ceratophyllum demersum* [35] and freshwater algae *Chlorella Pyrenoidosa* [36], which represents a type of photoprotective mechanism in plants. Therefore, the increased thermal dissipation may protect the chloroplasts from Cd stress when *P. crispus* was grown under blue light. Moreover, the rate of O_2_ exchange was not detectable in *P. crispus* grown in W + Cd and R + Cd, while it was about 2 mg O_2_ h^−1^ g^−1^ FW in *P. crispus* grown with B + Cd, indicating that Cd-treated *P. crispus* plants preserved their photosynthetic capacity when grown under blue light.

The photosynthesis of submerged macrophytes is often stressed by limited CO_2_ concentration underwater, which is due to the very low rates of CO_2_ diffusion in the aqueous environment and the external boundary layer that constrains the uptake of inorganic carbon [37]. Freshwater plants have evolved CO_2_-concentrating mechanisms (CCMs) in response to low CO_2_ stress [38]. HCO_3_^−^ utilization is one of the most frequent CCMs in freshwater macrophytes and is found in more than half of the tested species [37]. In this study, according to the results of pH-drift experiments, Cd-free-treated *P. crispus* plants were able to raise the end pH above 10, suggesting that they could utilize HCO_3_^−^ like other macrophytes during pH-drift experiments [38,39,40]. However, after *P. crispus* were exposed to Cd for 4 days, regardless of light quality, photosynthesis performed by *P. crispus* could not drive the pH to 9, and final CO_2_ concentrations in the solution were even kept at 25 μM (W + Cd), 12 μM (B + Cd), and 27 μM (R + Cd), respectively. Maberly (1990) has pointed out that the final pH could be used as an indicator to determine whether aquatic plants can utilize HCO_3_^−^; if the final pH is above 9, it indicates CO_2_ and HCO_3_^−^ use [41]. Thus, the present results indicate that HCO_3_^−^ acquisition in *P. crispus* was disturbed by Cd exposure, which is consistent with our previous reports on Cd-treated aquatic plant *O. alismoides* [27]. Additionally, C_T_/ALK is a judge for assessing the effectiveness of inorganic carbon (C_i_) depletion—a lower quotient means higher effectiveness of C_i_ utilization by plants [42]. In this study, C_T_/ALK was comparable and close to 1 among Cd-treated *P. crispus* regardless of light quality, suggesting that the C_i_ pool was nearly not utilized by Cd-exposed *P. crispus* plants. Recent studies have discovered the mechanisms of HCO_3_^−^ uptake in some aquatic plants [39,43]. It has been confirmed that extracellular carbonic anhydrase (CA_ext_) is vital for HCO_3_^−^ utilization in aquatic plants [39,40]. CA_ext_ converts HCO_3_^−^ to CO_2_ at the plasmalemma, and then CO_2_ diffuses into cells and is fixed by Rubisco. Previous studies have indicated that Cd can adversely affect HCO_3_^−^ utilization in photoautotrophs by inhibiting CA activity due to the replacement of CA’s active center by Cd [44]. Thus, Cd toxicity is likely to inhibit CA activity in *P. crispus*, decrease the conversion rate of HCO_3_^−^ to CO_2_, and eventually block the regular supply of CO_2_ to Rubisco. This resulted in a much lower utilization of HCO_3_^−^ in Cd-treated *P. crispus*.

Taken together, these results demonstrate that Cd induces phytotoxicity in *P. crispus*, and blue light enhances tolerance to Cd in this aquatic plant compared to red and white light. This is consistent with the findings of Guo et al. (2022) [23], who reported that blue light alleviated Cd-induced photosynthetic inhibition in cucumbers more effectively than red and white light [23]. The effects of light quality on plant growth and stress tolerance have been widely studied, with research indicating that different light qualities can impact plant tolerance to various types of abiotic stress. For example, studies have shown that the red-to-far-red light (R:FR) ratio can influence plant growth and stress tolerance, with low ratios being associated with improved photosynthetic efficiency and alleviated growth inhibition under stress conditions such as salt, cold, and calcium nitrate stress [45,46,47,48]. This suggests that light quality may play a role in regulating photosynthesis activity and subsequently affect plant stress tolerance. Guo et al. (2022) [23] speculated that red light, in contrast to blue light, increases the sensitivity of cucumbers to Cd toxicity by promoting Cd accumulation in a phytochrome B-dependent manner [23]. However, Gaion et al. (2017) [49] reported that phytochromes do not determine Cd stress tolerance in tomato plants [49]. Despite this discrepancy, additional research is necessary to elucidate the signaling pathway and specific mechanisms by which light quality regulates plant tolerance to Cd-induced phytotoxicity.

### 3.3. The Effect of Light Qualities on the Antioxidant System of P. crispus under Cd Stress

Cd-induced phytotoxicity is primarily caused by the excessive accumulation of reactive oxygen species (ROS) in plants [7,22]. These ROS are highly toxic and can cause significant damage to macromolecules, which can be reflected by the content of malondialdehyde (MDA) [50]. In this study, regardless of light quality, the significantly increased accumulation of MDA in Cd-treated *P. crispus* plants suggests that Cd exposure triggered the peroxidation of membrane lipids, indicating oxidative stress. This result is consistent with previous studies on Cd toxicity in plants [22,51]. Additionally, the MDA content in R + Cd-treated *P. crispus* plants was significantly higher than B + Cd-treated *P. crispus* plants, indicating that red light induced more severe oxidative stress in response to Cd exposure. Furthermore, Cd-stimulated overproduction of O_2_^.−^ in *P. crispus* leaves was verified by histochemical staining with NBT. Blue light visibly reduced O_2_^.−^ concentrations in *P. crispus* leaves under Cd stress compared to white and red light treatments. In summary, it is suggested that *P. crispus* treated with B + Cd suffered less oxidative stress.

Antioxidant enzymes are the main components of the antioxidant system in plants, which can eliminate ROS and protect cells from oxidative damage. SOD catalyzes O_2_^.−^ into H_2_O_2_, while CAT and APX further catalyze H_2_O_2_ decomposition [52]. GR catalyzes the reduction in GSSG to GSH to maintain the normal level of GSH in plants [53]. In this study, Cd stress led to a significant decrease in SOD, CAT, APX, and GR activity in *P. crispus*. Our findings are in line with previous research indicating that exposure to Cd stress can lead to a decrease in antioxidant capacity [54]. However, there have been studies that found an increase in SOD and CAT activities with an increase in Cd concentration, such as in strawberries [55]. In summary, our findings, along with data from the literature, suggest that Cd stress responses may not always be consistent, as they can vary depending on factors such as plant species and the severity of the stress induced by Cd. When compared to W + Cd, APX activity was significantly higher in B + Cd-grown *P. crispus*. The activation of specific enzymes is believed to be a key defense mechanism against oxidative stress resulting from exposure to toxic metals [56]. APX is a crucial enzyme that plays a significant role in the detoxification of H_2_O_2_ from cells. This enzyme provides tolerance to plants against free radicals, protecting them from oxidative stress caused by Cd [57,58]. Earlier studies have reported that Cd stress leads to elevated APX activity [59,60]. In the current study, we found that blue light-grown *P. crispus* had less pronounced Cd-induced oxidative stress. This suggests that blue light may enhance tolerance to Cd in *P. crispus* by activating antioxidant enzymes, particularly APX.

## 4. Materials and Methods

### 4.1. Plant Material and Pre-Treatment Culture Conditions

Healthy *P. crispus* plants were collected from the Wuhan Botanical Garden, Chinese Academy of Sciences. Prior to spectral and Cd treatments, the plants were acclimated for one week in 10% Hoagland’s solution [61] under ~100 μmol photon m^−2^ s^−1^ irradiation provided by white fluorescent tubes and 14 h of light per day in a growth room. After acclimation, uniform apical *P. crispus* shoots (~15 cm in length) were randomly selected and transplanted into 2 L plastic beakers (12 cm in diameter, 13 cm in height) for spectral and Cd treatments.

### 4.2. Spectral and Cd Treatments

**Figure 6:**
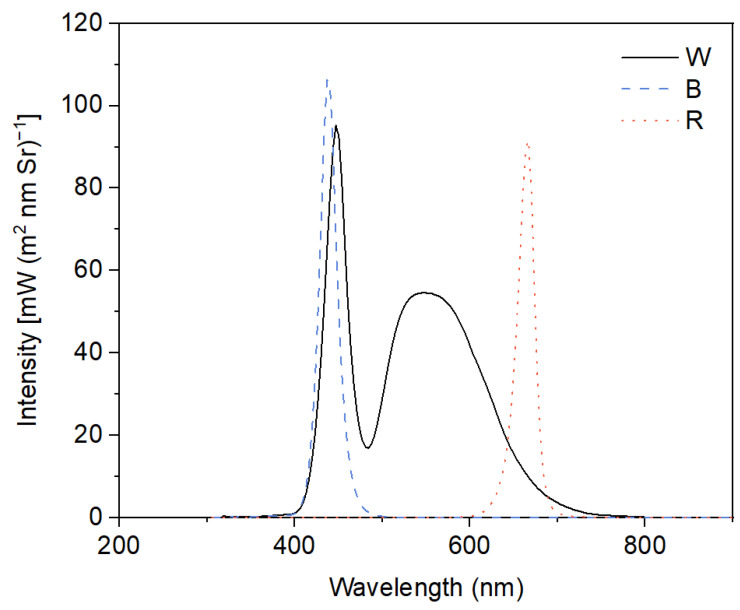
Light spectra in different light quality treatments. W, broad-spectrum white light provided by fluorescent tubes with peak emissions at 450 nm and 550 nm; B, monochromatic blue light provided by LED with a peak emission at 440 nm; R, monochromatic red light provided by LED with a peak emission at 665 nm.

In the present experiments, a 2 × 3 factorial design was used with two different concentrations of Cd (0 μM and 50 μM) and three light qualities (white light—W, blue light—B, and red light—R) as factors. This resulted in six treatments: W, W + Cd, B, B + Cd, R, and R + Cd. Cd^2+^ was provided by CdCl_2_·5H_2_O, and the concentration of Cd was determined based on pre-experimental results and our previous studies [9,22]. White fluorescent tubes (2019-I210 A1HC 6500 K, Foshan, China) were used to provide white light with a spectrum from 380 to 750 nm, with peaks at 450 and 550 nm. Monochromatic blue light with a peak at 440 nm and red light with a peak at 665 nm were provided by LED tubes (LH-T8 20 W-Z2, Lvheng, China). The spectrum of W, B, and R was detected by an underwater spectrometer (TriOS RAMSES, Germany) and is shown in Figure 6. The light intensity at the medium surface was maintained at 100 ± 10 μmol m^−2^ s^−1^, and the photoperiod was set to 14 h of light (08.00 h~22.00 h) and 10 h of darkness. There were four beakers of *P. crispus* plants for each treatment as replications, resulting in a total of 24 beakers of *P. crispus* plants treated in the present study. To prevent *P. crispus* plants from being irradiated by other light sources, compartments of the different light quality treatments were separated by plastic blackout curtains. The ambient temperature in each compartment was set to 25 ± 1 °C. After four days, *P. crispus* leaves from different treatments were collected and used for pigment content and chlorophyll fluorescence analysis, anatomical observation, histochemical detection of superoxide (O_2_^−^), and pH-drift experiments, as well as measurements of photosynthetic rate, antioxidant enzyme activity, and MDA content.

### 4.3. Observation of Leaf Anatomy and the Ultrastructure of Chloroplast and Mitochondria

The anatomic structure of *P. crispus* leaves and the ultrastructure of chloroplasts and mitochondria were studied according to previous methods [62]. For revealing the effect of different light qualities on the anatomic structure of *P. crispus* leaves with Cd treatment, semithin sections were obtained and observed with a light microscope (Motic BA310). For the examination of the ultrastructure of chloroplasts and mitochondria, ultrathin sections were observed with a transmission electron microscope (TEM) (Hitachi High-Tech, Tokyo, Japan).

### 4.4. Measurements of Pigment Content and Chlorophyll Fluorescence

Chlorophyll and carotenoids in the *P. crispus* leaf samples were extracted with 95% ethanol according to the previous method [63]. Chlorophyll fluorescence was determined with a Pulse-Amplitude-Modulation fluorometer (PAM 2500, Walz, Rohrdorf, Germany). Clean *P. crispus* leaf samples were kept in the dark and water for at least 15 min before measuring the maximum quantum yield of PSII (Fv/Fm). In addition to Fv/Fm, PamWin-3 software was used to record other chlorophyll fluorescence parameters including NPQ (non-photochemical quenching coefficient), Y(II) (effective quantum yield of PSII), and qP (photochemical quenching coefficient). The rapid light curves (RLCs) were also run with PAM 2500 to evaluate the influence of different light qualities on the photosynthetic efficiency and the state of the photosynthetic apparatus of *P. crispus* under Cd stress. The paired data (electron transport rate and photosynthetic active radiation) of RLCs were fitted according to the equations previously given by Platt et al. [64], and the resulting fitted parameters *ETR*_max_ (maximum relative electron transport rate) and α (initial slope of RCLs) were contained by referring to the research of Ralph and Gademann [65].

### 4.5. Measurement of Photosynthetic Rate

The photosynthetic rate of *P. crispus* leaves was measured according to our previous method with slight modification [13]. About 0.3~0.5 g FW of *P. crispus* leaf was gently sunk into the bottom of a Falcon tube (50 mL) with ~4 mm^3^ of sticky balls made by Blu-Tack to avoid the contact of leaf to the oxygen electrode (YSI Pro ODO Yellow Spring Instruments, USA), which was used to measure O_2_ concentration in the test solution. Tap water (with ~7.0 mg L^−1^ of initial dissolved O_2_) was used for the test solution, and the temperature was controlled at 25 ± 1 °C. During the tests, light quality and light intensity were the same as the treatment conditions.

### 4.6. pH-Drift Experiments

To investigate how light quality affects *P. crispus*’s ability to use HCO_3_^−^ under Cd stress, pH-drift experiments were conducted following the protocol of Maberly and Spence [42]. Approximately 0.2~0.3 g FW of *P. crispus* leaves collected from different treatments were incubated in 70 mL tightly sealed screw-cap plastic bottles with 50 mL of the test solution containing 500 μM NaHCO_3_ and 500 μM KHCO_3_. The sealed bottles with *P. crispus* leaves were exposed to corresponding light conditions (light quality and light intensity) that were consistent with the treatment conditions. After approximately 24 h of exposure, the leaves were removed from the bottles using a clean tweezer, and the final pH and alkalinity (ALK), as well as the concentration of inorganic carbon (CO_2_ and HCO_3_^−^) and C_T_/ALK of the medium, were measured and calculated using previously described methods [27,66].

### 4.7. Histochemical Detection of Superoxide (O_2_^.−^) and Measurements of Malondialdehyde (MDA) Content and Antioxidant Enzyme Activity

In situ O_2_^.−^ accumulations were detected by histochemical staining assays with nitroblue tetrazolium (NBT) according to Liu et al. [67]. The segments of the *P. crispus* leaf were stained in a 0.5 mg mL^−1^ NBT solution containing a 25 mM HEPES buffer (pH 7.8) at 25 °C in darkness for 2 h. Subsequently, the leaf segments were repeatedly rinsed in ethanol at 50~60 °C to completely remove the chlorophyll and were then photographed. The total content of MDA was detected following the thiobarbituric acid (TBA) method [68]. The activity of SOD, CAT, GR, and APX was determined with spectrophotometric assay kits (BC0170, BC0200, BC1160, and BC0220; Beijing Solarbio Science & Technology Co., Ltd., Beijing, China) by following the kit’s protocol and was expressed as U g^−1^ FW.

### 4.8. Statistical Analysis

Data in this study are presented as average ± SD. Independent sample *t*-tests and two-way analysis of variance (ANOVA) followed by Duncan’s and Tukey’s post-hoc tests were used to evaluate significant differences between treatments using SPSS 16.0 (SPSS Inc., Chicago, IL, USA). The significance level of the statistics was set at *p* < 0.05.

## 5. Conclusions

In summary, it can be concluded that Cd induced severe adverse morphological changes and decreased physicochemical properties in *P. crispus*, but the severity of the damage caused by Cd was less pronounced in blue light when compared to white and red light. Moreover, the lower level of MDA and less O_2_^.−^ implied that *P. crispus* grown under blue light suffered slighter oxidative stress compared to white and red light. The elevated APX activity and NPQ in leaves seemed to be closely related to the higher Cd tolerance in B-grown *P. crispus*. Further research is needed to elucidate the regulatory mechanisms of light quality and the possible involvement of photoreceptors in Cd detoxification in *P. crispus* plants. This study establishes a strong theoretical foundation for the future development of LED light control strategies aimed at regulating Cd uptake and tolerance in aquatic plants, particularly in severely polluted waters contaminated with heavy metals.

## Figures and Tables

**Table 1:** Summary of results of ANOVA (F-values and significance levels) for the effects of Cd and light quality and their interactions with *P. crispus* traits.

Item	Source		
	Cd	Light Quality	Cd × Light Quality
Chl a	3597.64 ***	6.19 **	12.80 ***
Chl b	1838.20 ***	2.77 ^NS^	7.01 **
Total Chl	3124.70 ***	5.10 *	11.27 **
Chl a/b	98.38 ***	0.93 ^NS^	1.00 ^NS^
Car	1612.05 ***	2.34 ^NS^	3.33 ^NS^
Chl/Car	129.58 ***	2.34 ^NS^	4.76 *
Fv/Fm	1036.18 ***	3.23 ^NS^	3.24 ^NS^
YⅡ	365.54 ***	2.87 ^NS^	0.97 ^NS^
qP	104.64 ***	12.74 **	5.20 *
NPQ	282.63 ***	0.86 ^NS^	3.19 ^NS^
ETR_max_	225.84 ***	0.96 ^NS^	0.77 ^NS^
α	934.50 ***	0.73 ^NS^	5.10 *
Final pH	797.54 ***	4.27 *	2.23 ^NS^
CO_2_	179.04 ***	1.01 ^NS^	1.01 ^NS^
HCO_3_^−^	1197.90 ***	1.17 ^NS^	1.18 ^NS^
C_T_/ALK	1720.08 ***	0.28 ^NS^	0.84 ^NS^
MDA	10.63 **	1.42 ^NS^	6.08 *
SOD	1188.38 ***	14.49 **	14.49 **
CAT	63.14 ***	8.84 **	6.66 *
GR	0.08 ^NS^	6.36 *	7.30 **
APX	419.65 ***	12.49 **	8.15 **

NS, not significant; * *p* < 0.05; ** *p* < 0.01; *** *p* < 0.001.

**Table 2:** The effect of light quality and Cd on the photosynthetic rate of *P. crispus*.

Treatments	O_2_ Evolution Rate (mg O_2_ h^−1^ g^−1^ FW)
W + Cd	No Detect
B + Cd	1.83 ± 0.79
R + Cd	No Detect

Date are presented as mean ± SD (*n* = 4). W + Cd, B + Cd, and R + Cd indicate the treatment of 50 µM Cd exposure under white light, 50 µM Cd exposure under blue light, and 50 µM Cd exposure under red light, respectively.

**Table 3:** Conditions and calculated carbon concentrations remaining at the end of the pH-drift experiments, with Cd-free or Cd-exposed *P. crispus* leaves grown under different light qualities for 4 days as drift materials.

Treatments	Final pH	ALK (equiv L^−1^)	C_T_ (mmol L^−1^)	CO_2_ (μmol L^−1^)	HCO_3_^−^ (mmol L^−1^)	CO_3_^2−^ (mmol L^−1^)	C_T_/ALK
W	10.64 ± 0.11 ^(a,α)^	1.00 ± 0.10 ^(a,α)^	0.17 ± 0.11 ^(a,α)^	0.002 ± 0.001 ^(a,α)^	0.04 ± 0.02 ^(a,α)^	0.14 ± 0.08 ^(a,α)^	0.17 ± 0.10 ^(a,α)^
W + Cd	7.90 ± 0.12 ^(AB,β)^	1.11 ± 0.12 ^(A,α)^	1.13 ± 0.13 ^(A,β)^	25.22 ± 9.97 ^(AB,β)^	1.10 ± 0.12 ^(A,β)^	0.01 ± 0.00 ^(A,β)^	1.01 ± 0.01 ^(A,β)^
B	10.73 ± 0.09 ^(a,α)^	1.04 ± 0.03 ^(a,α)^	0.11 ± 0.05 ^(a,α)^	0.001 ± 0.000 ^(a,α)^	0.04 ± 0.02 ^(a,α)^	0.16 ± 0.05 ^(a,α)^	0.10 ± 0.10 ^(a,α)^
B + Cd	8.36 ± 0.53 ^(A,β)^	1.06 ± 0.06 ^(A,α)^	1.03 ± 0.10 ^(A,β)^	12.12 ± 10.42 ^(A,β)^	0.99 ± 0.12 ^(A,β)^	0.01 ± 0.00 ^(A,β)^	0.97 ± 0.05 ^(A,β)^
R	10.66 ± 0.02 ^(a,α)^	1.04 ± 0.01 ^(a,α)^	0.18 ± 0.02 ^(a,α)^	0.001 ± 0.000 ^(a,α)^	0.04 ± 0.00 ^(a,α)^	0.15 ± 0.01 ^(a,α)^	0.18 ± 0.02 ^(a,α)^
R + Cd	7.84 ± 0.06 ^(B,β)^	1.06 ± 0.02 ^(A,α)^	1.08 ± 0.02 ^(A,β)^	26.97 ± 3.70 ^(B,β)^	1.04 ± 0.02 ^(A,β)^	0.01 ± 0.00 ^(A,β)^	1.02 ± 0.00 ^(A,β)^

Statistical differences are denoted with different lowercase letters among different light qualities without Cd treatment at the 0.05 level according to ANOVA followed by Duncan’s and Tukey’s post-hoc tests (*n* = 4). Statistical differences are denoted with different uppercase letters among different light qualities with Cd treatment at the 0.05 level according to ANOVA followed by Duncan’s and Tukey’s post-hoc tests (*n* = 4). A different Latin alphabet denotes statistical differences between Cd-free and Cd treatment under the same light quality at the 0.05 level according to independent sample *t*-tests (*n* = 4). W, W + Cd, B, B + Cd, R, and R + Cd indicate the treatment of no Cd under white light, 50 µM Cd exposure under white light, no Cd under blue light, 50 µM Cd exposure under blue light, no Cd under red light, and 50 µM Cd exposure under red light, respectively.

## Data Availability

All data are presented within the article.

## References

[1] Zhang Y.L., Jeppesen E., Liu X.H., Qin B.Q., Shi K., Zhou Y.Q., Thomaz S.M., Deng J.M. (2017). Global loss of aquatic vegetation in lakes. Earth-Sci. Rev..

[2] Ajiboye T.O., Oyewo O.A., Onwudiwe D.C. (2021). Simultaneous removal of organics and heavy metals from industrial wastewater: A review. Chemosphere.

[3] O’Hare M.T., Aguiar F.C., Asaeda T., Bakker E.S., Chambers P.A., Clayton J.S., Elger A., Ferreira T.M., Gross E.M., Gunn I.D.M. (2018). Plants in aquatic ecosystems: Current trends and future directions. Hydrobiologia.

[4] Mishra S., Srivastava S., Tripathi R.D., Govindarajan R., Kuriakose S.V., Prasad M.N.V. (2006). Phytochelatin synthesis and response of antioxidants during cadmium stress in *Bacopa monnieri* L.. Plant Physiol. Biochem..

[5] Clemens S. (2006). Toxic metal accumulation, response to exposure and mechanisms of tolerance in plants. Biochimie.

[6] Haider F.U., Cai L.Q., Coulter J.A., Cheema S.A., Wu J., Zhang R.Z., Ma W.J., Farooq M. (2021). Cadmium toxicity in plants: Impacts and remediation strategies. Ecotox. Environ. Safe..

[7] Berni R., Luyckx M., Xu X., Legay S., Sergeant K., Hausman J.F., Lutts S., Cai G., Guerriero G. (2019). Reactive oxygen species and heavy metal stress in plants: Impact on the cell wall and secondary metabolism. Environ. Exp. Bot..

[8] Sanità di Toppi L., Vurro E., Rossi L., Marabottini R., Musetti R., Careri M., Maffini M., Mucchino C., Corradini C., Badiani M. (2007). Different compensatory mechanisms in two metal-accumulating aquatic macrophytes exposed to acute cadmium stress in outdoor artificial lakes. Chemosphere.

[9] Huang W.M., Shao H., Zhou S.N., Zhou Q., Li W., Xing W. (2017). Modulation of cadmium-induced phytotoxicity in *Cabomba caroliniana* by urea involves photosynthetic metabolism and antioxidant status. Ecotox. Environ. Safe..

[10] Schutzendubel A., Polle A. (2002). Plant responses to abiotic stress: Heavy metal-induced oxidative stress and protection by mycorrhization. J. Exp. Bot..

[11] Asada K. (1992). Ascorbate peroxidase—A hydrogen peroxide-scavenging enzyme in plants. Physiol. Plant..

[12] Zhang Q.H., Dong X.H., Yang X.D., Liu E.F., Lin Q., Cheng L.J., Liu L., Jeppesen E. (2022). Aquatic macrophyte fluctuations since the 1900s in the third largest Chinese freshwater lake (Lake Taihu): Evidences, drivers and management implications. Catena.

[13] Wang L.Y., Han S.J., Wang S.W., Li W., Huang W.M. (2022). Morphological, photosynthetic, and CAM physiological responses of the submerged macrophyte *Ottelia alismoides* to light quality. Environ. Exp. Bot..

[14] Ilić Z.S., Fallik E. (2017). Light quality manipulation improves vegetable quality at harvest and postharvest: A review. Environ. Exp. Bot..

[15] Ahmadi T., Shabani L., Sabzalian M.R. (2019). Improvement in drought tolerance of lemon balm, *Melissa officinalis* L. under the pre-treatment of LED lighting. Plant Physiol. Biochem..

[16] Kirk J.T.O. (2011). Light and Photosynthesis in Aquatic Ecosystems.

[17] Trouwborst G., Hogewoning S.W., van Kooten O., Harbinson J., van Ieperen W. (2016). Plasticity of photosynthesis after the ‘red light syndrome’ in cucumber. Environ. Exp. Bot..

[18] Tehrani P.F., Majd A., Mahmoodzadeh H., Satari T.N. (2016). Effect of red and blue light–emitting diodes on germination, morphological and anatomical features of *Brassica napus*. Adv. Stud. Biol..

[19] Pettai H., Oja V., Freiberg A., Laisk A. (2005). Photosynthetic activity of far-red light in green plants. BBA–Bioenerg..

[20] Muneer S., Kim E.J., Park J.S., Lee J.H. (2014). Influence of green, red and blue light emitting diodes on multiprotein complex proteins and photosynthetic activity under different light intensities in lettuce leaves (*Lactuca sativa* L.). Int. J. Mol. Sci..

[21] Li J.F., Yi C.Y., Zhang C.R., Pan F., Xie C., Zhou W.Z., Zhou C.F. (2021). Effects of light quality on leaf growth and photosynthetic fluorescence of *Brasenia schreberi* seedlings. Heliyon.

[22] Huang W.M., Han S.J., Zhou Q., Li W., Xing W. (2019). Assessing interactions between environmental factors and aquatic toxicity: Influences of dissolved CO_2_ and light on Cd toxicity in the aquatic macrophyte *Potamogeton crispus*. Aquat. Toxicol..

[23] Guo Z.X., Lv J.L., Zhang H.M., Hu C.Y., Qin Y.P., Dong H., Zhang T., Dong X.X., Du N.S., Piao F.Z. (2022). Red and blue light function antagonistically to regulate cadmium tolerance by modulating the photosynthesis, antioxidant defense system and Cd uptake in cucumber (*Cucumis sativus* L.). J. Hazard. Mater..

[24] Xu Q.S., Min H.L., Cai S.J., Fu Y.Y., Sha S., Xie K.B., Du K.H. (2012). Subcellular distribution and toxicity of cadmium in *Potamogeton crispus* L.. Chemosphere.

[25] Sivaci A., Elmas E., Gümüş F. (2008). Removal of cadmium by *Myriophyllum heterophyllum* Michx. and *Potamogeton crispus* L. and its effect on pigments and total phenolic compounds. Arch. Environ. Contam. Toxicol..

[26] Artetxe U., García-Plazaola J.I., Hernández A., Becerril J.M. (2002). Low light grown duckweed plants are more protected against the toxicity induced by Zn and Cd. Plant Physiol. Bioch..

[27] Huang W.M., Jin Q., Yin L.Y., Li W. (2020). Responses of CO_2_-concentrating mechanisms and photosynthetic characteristics in aquatic plant *Ottelia alismoides* following cadmium stress under low CO_2_. Ecotox. Environ. Safe..

[28] Zeshan A., Abdullah M., Adil M.F., Wei D.M., Noman M., Ahmed T., Sehar S., Ouyang Y.N., Shamsi I.H. (2022). Improvement of morpho-physiological, ultrastructural and nutritional profiles in wheat seedlings through astaxanthin nanoparticles alleviating the cadmium toxicity. J. Hazard. Mater..

[29] Zhang P., Wang R., Ju Q., Li W., Lam-Son P.T., Xu J. (2019). The R2R3-MYB transcription factor MYB49 regulates cadmium accumulation. Plant Physiol..

[30] Buchanan B.B., Gruissem W., Jones R.L. (2000). Biochemistry & Molecular Biology of Plants.

[31] Neves N.R., Oliva M.A., Centeno D.C., Costa A.C., Ribas R.F., Pereira E.G. (2009). Photosynthesis and oxidative stress in the resting plant species *Eugenia uniflora* L. exposed to simulated acid rain and iron ore dust deposition: Potential use in environmental risk assessment. Sci. Total Environ..

[32] Singh S., Parihar P., Singh R., Singh V.P., Prasad S.M. (2016). Heavy metal tolerance in plants: Role of transcriptomics, proteomics, metabolomics, and ionomics. Front. Plant Sci..

[33] Dobrikova A.G., Apostolova E.L., Hanć A., Yotsova E., Borisova P., Sperdouli I., Adamakis I.D.S., Moustakase M. (2021). Cadmium toxicity in *Salvia sclarea* L.: An integrative response of element uptake, oxidative stress markers, leaf structure and photosynthesis. Ecotox. Environ. Safe..

[34] Marchello A.E., Oliveira N.L., Lombardi A.T., Polpo A. (2018). An investigation onto Cd toxicity to freshwater microalga *Chlorella sorokiniana* in mixotrophy and photoautotrophy: A Bayesian approach. Chemosphere.

[35] Andresen E., Kappel S., Stärk H.J., Riegger U., Borovec J., Mattusch J., Heinz A., Schmelzer C.E., Matoušková Š., Dickinson B. (2016). Cadmium toxicity investigated at the physiological and biophysical levels under environmentally relevant conditions using the aquatic model plant *Ceratophyllum demersum*. New Phytol..

[36] Wang S., Wufuer R., Duo J., Li W., Pan X. (2022). Cadmium caused different toxicity to photosystem I and photosystem II of freshwater unicellular algae *Chlorella pyrenoidosa* (Chlorophyta). Toxics.

[37] Iversen L.L., Winkel A., Baastrup-Spohr L., Hinke A.B., Alahuhta J., Baattrup-Pedersen A., Birk S., Brodersen P., Chambers P.A., Ecke F. (2019). Catchment properties and the photosynthetic trait composition of freshwater plant communities. Science.

[38] Maberly S.C., Gontero B. (2017). Ecological imperatives for aquatic CO_2_-concentrating mechanisms. J. Exp. Bot..

[39] Huang W.M., Han S.J., Jiang H.S., Gu S.P., Li W., Gontero B., Maberly S.C. (2020). External α-carbonic anhydrase and solute carrier 4 are required for bicarbonate uptake in a freshwater angiosperm. J. Exp. Bot..

[40] Wang S.N., Li P.P., Liao Z.Y., Wang W.W., Chen T., Yin L.Y., Jiang H.S., Li W. (2022). Adaptation of inorganic carbon utilization strategies in submerged and floating leaves of heteroblastic plant *Ottelia cordata*. Environ. Exp. Bot..

[41] Maberly S.C. (1990). Exogenous sources of inorganic carbon for photosynthesis by marine macroalgae. J. Phycol..

[42] Maberly S.C., Spence D.H.N. (1983). Photosynthetic inorganic carbon use by freshwater plants. J. Ecol..

[43] Maberly S.C., Gontero B., Adams W.W., Terashima I. (2018). Trade-offs and synergies in the structural and functional characteristics of leaves photosynthesizing in aquatic environments. The Leaf: A Platform for Performing Photosynthesis.

[44] Tóth T., Zsiros O., Kis M., Garab G., Kovacs L. (2012). Cadmium exerts its toxic effects on photosynthesis via a cascade mechanism in the cyanobacterium, *Synechocystis* PCC 6803. Plant Cell Environ..

[45] Cao K., Jie Y., Xu D., Ai K., Bao E., Zou Z. (2018). Exposure to lower red to far-red light ratios improve tomato tolerance to salt stress. BMC Plant Biol..

[46] Wang F., Zhang L., Chen X., Wu X., Xiang X., Zhou J., Xia X., Shi K., Yu J., Foyer C.H. (2018). SlHY5 integrates temperature, light, and hormone signaling to balance plant growth and cold tolerance. Plant Physiol..

[47] Zhou X.T., Li Z.L., He J.J., Wang X.Y., Liu Q.L., Huang J., Xie Y.D., He Z.Q. (2021). Effects of red to far-red light ratio on growth and photosynthetic characteristics of tomato seedlings under calcium nitrate stress. Photosynthetica.

[48] Chen L.B., Huang J., Liu Q.L., Li Z.L., Chen X., Han J.X., Gan Y.R., He Y.X., Jiang C.X., Tang Y.X. (2022). Low R/FR ratio affects Pakchos growth and nitrate content under excess nitrate stress. Horticulturae.

[49] Gaion L.A., Lorevice P.G., Monteiro C.C., Gavassi M.A., D’Amico-Damião V., Gratão P.L., Gasparino E.C., Carvalho R.F. (2017). The role of phytochromes in cadmium stress responses in tomato. Bragantia.

[50] Sidhu G.P.S., Singh H.P., Batish D.R., Kohli R.K. (2017). Tolerance and hyper-accumulation of cadmium by a wild, unpalatable herb *Coronopus didymus* (L.) Sm. (*Brassicaceae*). Ecotox. Environ. Safe..

[51] Jia X., Zhao Y.H., Liu T., He Y.H. (2017). Leaf defense system of *Robinia pseudoacacia* L. seedlings exposed to 3 years of elevated atmospheric CO_2_ and Cd-contaminated soils. Sci. Total Environ..

[52] Neill S.J., Desikan R., Clarke A., Hurst R.D., Hancock J.T. (2002). Hydrogen peroxide and nitric oxide as signalling molecules in plants. J. Exp. Bot..

[53] Noctor G., Mhamdi A., Chaouch S., Han Y., Neukermans J., Marquez-Garcia B., Queval G., Foyer C.H. (2012). Glutathione in plants: An integrated overview. Plant Cell Environ..

[54] Głowacka K., Źróbek-Sokolnik A., Okorski A., Najdzion J. (2019). The effect of cadmium on the activity of stress-related enzymes and the ultrastructure of Pea Roots. Plants.

[55] Muradoglu F., Gundogdu M., Ercisli S., Encu T., Balta F., Jaafar H.Z., Zia-Ul-Haq M. (2015). Cadmium toxicity affects chlorophyll a and b content, antioxidant enzyme activities and mineral nutrient accumulation in strawberry. Biol. Res..

[56] Dat J., Vandenabeele S., Vranov E., van Montagu M., Inze D., van Breusegem F. (2000). Dual action of the active oxygen species during plant stress responses. Cell Mol. Life Sci..

[57] Ramzan M., Ayub F., Shah A.A., Naz G., Shah A.N., Malik A., Sardar R., Telesiński A., Kalaji H.M., Dessoky E.S. (2022). Synergistic effect of zinc oxide nanoparticles and *Moringa oleifera* leaf extract alleviates cadmium toxicity in *Linum usitatissimum*: Antioxidants and physiochemical studies. Front. Plant Sci..

[58] Metwally A., Finkemeier I., Georgi M., Dietz K.J. (2003). Salicylic acid alleviates the cadmium toxicity in barley seedlings. Plant Physiol..

[59] Shaw P. (1995). Effects of mercury and cadmium on the activities of antioxidative enzymes in the seedlings of *Phaseolus aureus*. Biol. Plant.

[60] Ali M.B., Chun H.S., Kim B.K., Lee C.B. (2002). Cadmium-induced changes in antioxidant enzyme activities in rice (*Oryza sativa* L. cv. Dongjin). J. Plant Biol..

[61] Hoagland D.R., Arnon D.I. (1950). The water-culture method for growing plants without soil. Circ. Calif. Agric. Exp. Stn..

[62] Han S.J., Maberly S.C., Gontero B., Xing Z.F., Jiang H.S., Huang W.M. (2020). Structural basis for C_4_ photosynthesis without kranz anatomy in leaves of the submerged freshwater plant *Ottelia alismoides*. Ann. Bot..

[63] Brain R.A., Solomon K.R. (2007). A protocol for conducting 7-day daily renewal tests with *Lemna gibba*. Nat. Protoc..

[64] Platt T., Gallegos C.L., Harrison W.G. (1980). Photoinhibition of photosynthesis in natural assemblages of marine phytoplankton. J. Mar. Res..

[65] Ralph P.J., Gademann R. (2005). Rapid light curves: A powerful tool to assess photosynthetic activity. Aquat. Bot..

[66] Maberly S.C. (1996). Diel, episodic and seasonal changes in pH and concentrations of inorganic carbon in a productive lake. Freshwater Biol..

[67] Liu N., Jin Z.Y., Wang S.S., Gong B., Wen D., Wang X.F., Wei M., Shi Q.H. (2015). Sodic alkaline stress mitigation with exogenous melatonin involves reactive oxygen metabolism and ion homeostasis in tomato. Sci. Hortic..

[68] Heath R.L., Packer L. (1968). Photoperoxidation in isolated chloroplasts: I. Kinetics and stoichiometry of fatty acid peroxidation. Arch. Biochem. Biophys..

